# Health Economic Analysis of Two-Layer Bandage System for Treatment of Chronic Venous Insufficiency

**DOI:** 10.36469/001c.82159

**Published:** 2023-08-25

**Authors:** Peter J. Mallow

**Affiliations:** 1 Xavier University, Cincinnati, OH, USA

**Keywords:** chronic venous insufficiency, compression therapy, health economics, two-layer bandage system

## Abstract

**Background:** Compression therapy is the gold standard for the treatment of chronic venous insufficiency (CVI). Two-layer bandage (2LB) systems have been shown to be a safe and effective treatment option.

**Objective:** To estimate the total cost per response (CPR) for the resolution of edema and wounds in patients with CVI treated with a 2LB system as part of their overall wound healing regimen.

**Methods:** A probabilistic decision tree model was developed to estimate the incremental CPR for a 2LB system. The model simulated 10 000 patients to estimate the CPR for the resolution of edema and wound healing. The analysis was performed using clinical data from a published single-arm, multicenter prospective study of CVI indicated for compression therapy. The response outcomes of interest were resolution of edema and rate of wound healing. The follow-up time was a maximum of 6 weeks, and the perspective of the study was a US outpatient treatment center. Economic data for compression therapy were based on the public prices of a 2LB system. Dressing changes occurred per manufacturer instructions for use.

**Results:** The study comprised 702 patients (56% female), with a total of 414 wounds. The median duration of the wounds was 42 days, and the median size at the initial visit was 3.5 cm2. The average pain reduction fell by 67% using a visual analog score. Bandages were typically changed once or twice a week (51.7%). Wound healing occurred in 128 of the 414 wounds (30.9%). The expected incremental CPR of a 2LB system for the resolution of edema was 65.67(range,16.67-124.32).TheexpectedincrementalCPRofa2LBsystemforthehealingofawoundwas138.71 (range, 35.71−273.53).

**Conclusion:** This economic evaluation complements previous clinical effectiveness and safety studies of 2LB systems for the treatment of CVI. The results demonstrate that the costs of incorporating 2LB into standard wound-healing protocols are negligible compared with overall treatment costs. Two-layer bandages may be considered a cost-effective first-line system for the treatment of wounds caused by CVI.

## BACKGROUND

Chronic venous insufficiency (CVI) is common in the United States and occurs in up to 40% of the population.^1–3^ CVI affects the venous system and manifests as lower-extremity edema, pain, and, in severe cases, ulcers.^4^ Untreated or poor management of CVI leads to a decrease in the patient’s quality of life and interferes with activities of daily living.^5^ The healthcare costs of a conservative treatment regimen, including ulcers and edema, on average exceed $2400 per month, or more than $5500 per episode.^6–8^ Collectively, US healthcare costs for CVI exceed $4.9 billion annually.^9^

The use of compression therapy is part of the standard treatment protocol for CVI.^10–12^ A novel 2-layer bandage system (2LB) has been shown to be safe, efficacious, and well-tolerated by patients for the treatment of CVI.^13–18^ However, there is limited economic evidence regarding the use of a 2LB system. The objective of the study was to estimate the total cost per response (CPR) for edema resolution and wound healing of patients with CVI treated with a 2LB system as part of their overall treatment regimen.

## METHODS

### Clinical Data

The clinical data were obtained from a recently published, prospective, multicenter study.^19^ This study recruited all CVI patients presenting with a venous leg ulcer or edema in which the physician made the clinical judgment to treat with a compression system. A 2LB system was used per the manufacturer’s instructions for use, and all treatment decisions were made by the patient’s physician. Patients were followed in an outpatient setting for a maximum of 6 weeks (average, 27 days). All patients were treated with a 2LB system for the duration of the study. The 2LB system components were a padded inelastic bandage with short elongation and an elastic bandage with lengthy elongation. The system provides a visual indicator to facilitate the proper application and achieve the desired pressure. The system can be worn 24 hours a day for up to 7 days per the instructions for use.^13^

The study comprised a total of 702 patients (56% female) and included 491 cases of edema and 414 wounds. Bandages were typically changed 1 or 2 times a week (51.7%). For those with wounds, the median duration of the wounds was 42 days, and the median size at the initial visit was 3.5 cm^2^. Wound healing occurred in 128 of the 414 wounds (30.9%) within the 6-week study period. The proportion of patients with edema that resolved at the end of the study period was 67.1%. All data were publicly available, and the study was deemed exempt from institutional review board review.

### Cost and Utilization Data

The cost and utilization data were obtained from a pragmatic literature review. A search of English-language articles from January 2019 to January 2023 was performed in PubMed and Google Scholar for the model parameters. The articles were reviewed based on applicability, recency, and quality by a single reviewer. In the event of more than one study meeting the criteria, a pooled analysis was applied to identify the midpoint value used for the analysis. The cost and utilization data consisted of US national average patient-level data to allow for the results to be applied to a typical health system.

### Modeling Strategy

**Figure 1.**
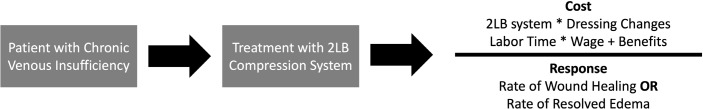
Model Diagram The model framework was for an individual patient over a 6-week period. The model perspective was an outpatient treatment center in the United States. Discounting was not applied due to the short time horizon. A total of 10 000 patients were simulated to generate the results. Abbreviation: 2LB, two-layer bandage.

A patient-level probabilistic decision tree model was developed to estimate the incremental CPR for the 2LB system (**Figure 1**). The perspective of the model was an outpatient treatment center in the United States. The cost parameters included the cost of the 2LB system and the medical technician’s time to apply the 2LB. The outcome measures were the percentage of edema resolved and the percentage of wounds healed at the conclusion of the follow-up period. The CPR was calculated with the following formula for edema resolved and wound healed:

$$\text{CPR}=\sum {\text{C}}_{\text{2LB}}/{\text{Outcome}}_{\text{2LB}}$$

where C_2LB_ is the summation of the costs of the 2LB system, and Outcome_2LB_ is either the percentage of edema resolved or wound healed. Each model was performed separately, with the only difference being the outcome variable. A total of 10 000 patients were simulated. The base case model values were varied based on the reported ranges shown in **Table 1**. The models were developed using Treeage Software (Williamstown, Massachusetts). All data used in the model were publicly available in the literature.

**Table 1.** Model Parameters

**Parameter**	**Base Case**	**Sensitivity Analysis**		**Distribution**	**Reference**
		**Low**	**High**		
Cost of 2LB system ($)	12.00	9.00	15.00	Uniform	Urgo Medical^20^
No. of dressing changes, over study period	4	1	6	Uniform	Chakravarthy et al^21^
Time to change 2LB (sec)^a^	65	59	71	Uniform	Chakravarthy et al^21^
Hourly medical technician wage ($)	26.00	26.00	26.00	Fixed	Chakravarthy et al^21^
Resolved edema (%)	67	60	74	Triangle	Stucker et al^19^
Wound healed (%)	31	28	34	Triangle	Stucker et al^19^

^a^Time to change 2LB system did not include wound dressings.

Abbreviation: 2LB, two-layer bandage.

## RESULTS

**Figure 2.**
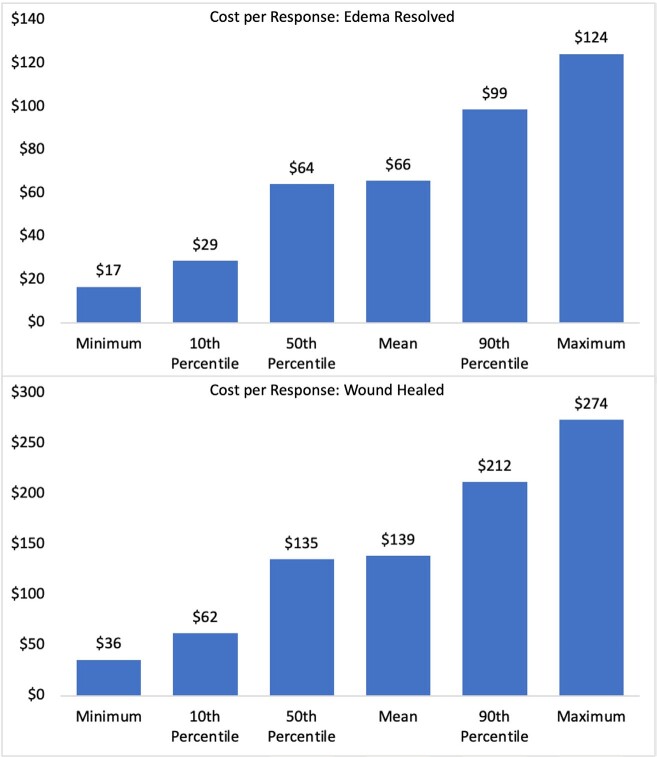
Cost per Response for Edema Resolved and Wound Healed

**Figure 2** (top panel) and **Table 2** show the probabilistic model results to resolve edema using the 2LB system. The mean expected incremental CPR was $65.67. Based on 10 000 simulated patients, the cost to resolve edema ranged from $16.67 to $124.32. The associated cost of the 2LB system over the study period ranged from $10.00 to $92.00, and the probability of resolving the edema ranged from 60% to 74%.

**Table 2.** Probabilistic Model Results: Edema Resolved

	**Cost ($)**	**Probability of Edema Resolved (%)**	**Cost per Wound Healed ($)**
Mean	44.00	67	65.67
Minimum	10.00	60	16.67
10th percentile	18.00	63	28.57
50th percentile	43.00	67	64.18
90th percentile	70.00	71	98.59
Maximum	92.00	74	124.32

**Figure 2** (lower panel) and **Table 3** show the probabilistic model results in healing one wound. After 10 000 simulated patient trials, the mean expected incremental CPR was $138.71 and ranged from $35.71 to $273.53. The associated cost of the 2LB system over the study period ranged from $10.00 to $92.00, and the probability of healing the wound ranged from 28% to 34%.

**Table 3.** Probabilistic Model Results: Wound Healed

	**Cost ($)**	**Probability of Wound Healed (%)**	**Cost per Wound Healed ($)**
Mean	43.00	31	138.71
Minimum	10.00	28	35.71
10th percentile	18.00	29	62.07
50th percentile	42.00	31	135.48
90th percentile	70.00	33	212.12
Maximum	93.00	34	273.53

## DISCUSSION

This study found the CPR for edema resolved or wound healed was expected to be approximately $66 or $139, respectively. The CPR approach is an effective means to evaluate an intervention because it combines the additional costs of the intervention, 2LB, with the benefit provided by the intervention. The result is more indicative of the overall costs after adjusting for patients who do not benefit from the intervention.

The overall costs to treat CVI run in excess of $5500.^6–8^ Thus, after adjusting for treatment failures, the use of a 2LB system was expected to account for 1% of the total cost to resolve edema or 3% of healing a wound. This cost is negligible in the overall treatment costs of patients with CVI.

The insignificant cost of the 2LB system should emphasize the safety, efficacy, and patient satisfaction of the 2LB system. A randomized controlled trial of a 2LB system and 4-layer bandage (4LB) system found that 2LB was safe and as effective as the 4LB and easier to apply.^13–15^ Appropriate pressure has been shown to be maintained with the 2LB system.^16,22–24^ Patient satisfaction with the 2LB system was 70% compared with 12% with the 4LB system, which led to increased compliance with the 2LB system.^22^ Finally, the average pain reduction fell by 67% using a visual analog score.^19^

### Limitations

The results of this study were based on clinical data from a noncomparative observational study. Therefore, comparisons to other bandage systems were not possible. However, this study was a large prospective, multicenter study, which provided a real-world setting to conduct an economic evaluation. Second, this study examined the incremental costs of adding a 2LB system into the standard CVI treatment protocol. It was not intended to provide a total cost to treat a patient with CVI. Finally, this study was conducted from an outpatient clinic perspective and may not be generalizable to other sites of care.

## CONCLUSION

This study provides economic evidence for using a 2LB system for the treatment of CVI in the outpatient setting. This study complements previous studies demonstrating the efficacy, safety, and increased patient satisfaction of a 2LB system. The use of a 2LB system should be considered an added economic value to the management of CVI.

### Disclosures

The author has a consulting relationship with Urgo Medical North America (UMNA).

### Ethics Approval and Data Availability

This research used publicly available secondary data.

## References

[ref-222208] Beebe-Dimmer Jennifer L., Pfeifer John R., Engle Jennifer S., Schottenfeld David (2005). The epidemiology of chronic venous insufficiency and varicose veins. Annals of Epidemiology.

[ref-222209] Evans C. J., Fowkes F. G., Ruckley C. V., Lee A. J. (1999). Prevalence of varicose veins and chronic venous insufficiency in men and women in the general population: Edinburgh Vein Study. Journal of Epidemiology & Community Health.

[ref-222210] Kim Young, Maximilian Png C.Y., Sumpio Brandon J., DeCarlo Charles S., Dua Anahita (2021). Defining the human and health care costs of chronic venous insufficiency. Seminars in Vascular Surgery.

[ref-222211] Porter John M., Moneta Gregory L. (1995). Reporting standards in venous disease: an update. International Consensus Committee on Chronic Venous Disease. Journal of Vascular Surgery.

[ref-222212] Enden Tone, Wik Hilde Skuterud, Kvam Ann Kristin, Haig Ylva, Kløw Nils Einar, Sandset Per Morten (2013). Health-related quality of life after catheter-directed thrombolysis for deep vein thrombosis: secondary outcomes of the randomised, non-blinded, parallel-group CaVenT study. BMJ Open.

[ref-222213] Raffetto Joseph D., Ligi Daniela, Maniscalco Rosanna, Khalil Raouf A., Mannello Ferdinando (2021). Why venous leg ulcers have difficulty healing: overview on pathophysiology, clinical consequences, and treatment. Journal of Clinical Medicine.

[ref-222214] Melikian Raffi, O'Donnell Thomas F., Jr., Iafrati Mark (2022). The economic impact of infection requiring hospitalization on venous leg ulcers. Journal of Vascular Surgery: Venous and Lymphatic Disorders.

[ref-222215] Olin Jeffrey W, Beusterien Kathleen M, Childs Mary Beth, Seavey Caroline, McHugh Linda, Griffiths Robert I (1999). Medical costs of treating venous stasis ulcers: evidence from a retrospective cohort study. Vascular Medicine.

[ref-222216] Kolluri Raghu, Lugli Marzia, Villalba Laurencia, Varcoe Ramon, Maleti Oscar, Gallardo Fernando, Black Stephen, Forgues Fannie, Lichtenberg Michael, Hinahara Jordan, Ramakrishnan Saranya, Beckman Joshua A. (2022). An estimate of the economic burden of venous leg ulcers associated with deep venous disease. Vascular Medicine.

[ref-222217] Bolton L.L., Girolami S., Corbett L., van Rijswijk L. (2014). The Association for the Advancement of Wound Care (AAWC) venous and pressure ulcer guidelines. Ostomy Wound Manage.

[ref-222218] Franks Peter J, Barker Judith, Collier Mark, Gethin Georgina, Haesler Emily, Jawien Arkadiusz, Laeuchli Severin, Mosti Giovanni, Probst Sebastian, Weller Carolina (2016). Management of patients with venous leg ulcers: challenges and current best practice. Journal of Wound Care.

[ref-222219] O’Donnell Thomas F., Jr., Passman Marc A., Marston William A., Ennis William J., Dalsing Michael, Kistner Robert L., Lurie Fedor, Henke Peter K., Gloviczki Monika L., Eklöf Bo G., Stoughton Julianne, Raju Sesadri, Shortell Cynthia K., Raffetto Joseph D., Partsch Hugo, Pounds Lori C., Cummings Mary E., Gillespie David L., McLafferty Robert B., Murad Mohammad Hassan, Wakefield Thomas W., Gloviczki Peter (2014). Management of venous leg ulcers: clinical practice guidelines of the Society for Vascular Surgery and the American Venous Forum. Journal of Vascular Surgery.

[ref-222220] Lazareth I., Moffatt C., Dissemond J., Padieu A.S. Lesne, Truchetet F., Beissert S., Wicks G., Tilbe H., Sauvadet A., Bohbot S., Meaume S. (2012). Efficacy of two compression systems in the management of VLUs: results of a European RCT. Journal of Wound Care.

[ref-222221] Jünger M., Ladwig A., Bohbot S., Haase H. (2009). Comparison of interface pressures of three compression bandaging systems used on healthy volunteers. Journal of Wound Care.

[ref-222222] Hanna Richard, Bohbot Serge, Connolly Nicki (2008). A comparison of interface pressures of three compression bandage systems. British Journal of Nursing.

[ref-222223] Benigni J.P, Lazareth I, Parpex P., Gerard J.L, Alves M, Vin F, Meaume S, Senet P, Allaert F.A, Sauvadet A, Bohbot S (2007). Efficacy, safety and acceptability of a new two-layer bandage system for venous leg ulcers. Journal of Wound Care.

[ref-222224] Tai Hong Qian, Chaen Lester Chong Rhan, Boey Johnson, Kime Sally, Rial Rodrigo, Montero Elena Conde, Atkin Leanne, Stansal Audrey, Isabelle Lazareth, Tickner Anthony, Vlad Lucian G, Lantis John, Hester Colboc, Galea Emilio (2021). A dual pressure indicator, two-layer compression system for the treatment of venous leg ulcers: a review. Journal of Wound Care.

[ref-222225] Goka E.A., Poku E., Thokala P., Sutton A. (2020). Clinical and economic impact of two-layer compression system for the treatment of venous leg ulcers: a systematic review. Wounds.

[ref-222226] Stucker Markus, Munter Karl-Christian, Erfurt-Berge Cornelia, Lützkendorf Steffen, Eder Stephan, Möller Udo, Dissemond Joachim (2021). Multicomponent compression system use in patients with chronic venous insufficiency: a real-life prospective study. Journal of Wound Care.

[ref-222230] Medical Urgo International guidelines recognize the superior efficacy of multi-component bandages.

[ref-222231] Chakravarthy D., Mallow P., Pilati L. The health economic implications of a comparative time and motion study done on two bandage systems, one traditional four layer system, the other a novel two layer dual compression system (DCS).

[ref-222227] Pilati L., Houserman D. (2020). Comparing 2-layer with traditional 4-layer compression therapy. Wound Manag Prev.

[ref-222228] Greenstein E., Tickner A. (2020). Addressing compression continuity, consistency, and comfort using a dual compression system. Wound Manag Prev.

[ref-222229] Lantis John C., II, Barrett Christopher, Couch Kara S, Ehmann Suzie, Greenstein Emily, Ostler Marta, Tickner Anthony (2020). A dual compression system: preliminary clinical insights from the US. Journal of Wound Care.

